# Activity-Based Protein Profiling Reveals Mitochondrial Oxidative Enzyme Impairment and Restoration in Diet-Induced Obese Mice

**DOI:** 10.1371/journal.pone.0047996

**Published:** 2012-10-24

**Authors:** Natalie C. Sadler, Thomas E. Angel, Michael P. Lewis, LeeAnna M. Pederson, Lacie M. Chauvigné-Hines, Susan D. Wiedner, Erika M. Zink, Richard D. Smith, Aaron T. Wright

**Affiliations:** Biological Sciences Division, Pacific Northwest National Laboratory, Richland, Washington, United States of America; College of Tropical Agriculture and Human Resources, University of Hawaii, United States of America

## Abstract

High-fat diet (HFD) induced obesity and concomitant development of insulin resistance (IR) and type 2 diabetes mellitus have been linked to mitochondrial dysfunction. However, it is not clear whether mitochondrial dysfunction is a direct effect of a HFD, or if mitochondrial function is reduced with increased HFD duration. We hypothesized that the function of mitochondrial oxidative and lipid metabolism functions in skeletal muscle mitochondria for HFD mice are similar, or elevated, relative to standard diet (SD) mice; thereby, IR is neither cause nor consequence of mitochondrial dysfunction. We applied a chemical probe approach to identify functionally reactive ATPases and nucleotide-binding proteins in mitochondria isolated from skeletal muscle of C57Bl/6J mice fed HFD or SD chow for 2-, 8-, or 16-weeks; feeding time points known to induce IR. A total of 293 probe-labeled proteins were identified by mass spectrometry-based proteomics, of which 54 differed in abundance between HFD and SD mice. We found proteins associated with the TCA cycle, oxidative phosphorylation (OXPHOS), and lipid metabolism were altered in function when comparing SD to HFD fed mice at 2-weeks, however by 16-weeks HFD mice had TCA cycle, β-oxidation, and respiratory chain function at levels similar to or higher than SD mice.

## Introduction

Type 2 diabetes mellitus (T2DM) is a prevalent metabolic disease [1], and its etiology involves complex interactions between genetic and environmental factors including physical inactivity, increased caloric intake, and obesity [2]. Despite the rates of incidence and massive costs associated with treating T2DM, significant questions remain about the development of this disease.

Skeletal muscle insulin resistance (IR) is an early and important event in the pathogenesis of obesity and T2DM. Insulin resistance is defined as a relative impairment in the ability of insulin to exert its effects on glucose and lipid metabolism in target tissues [3]. Despite significant efforts, key questions remain: do high-fat diets and obesity result in mitochondrial dysfunction, what role in the pathophysiology of IR and T2DM does skeletal muscle mitochondrial dysfunction play, and is mitochondrial dysfunction a cause or consequence of IR and T2DM [3], [4], [5], [6]?

Human and rodent studies of mitochondrial dysfunction as a result of obesity, and progenitor or effect of IR and T2DM, provide conflicting views. In humans, studies have shown the down-regulation of genes encoding mitochondrial enzymes [7], [8], decreased mitochondrial density [9], and lower respiratory chain activity [9], but other studies found no mitochondrial dysfunction in insulin resistant skeletal muscle [10], [11], [12], [13]. It was shown that levels of both mitochondrial oxidative phosphorylation and electron transport capacity in obese women and T2DM patients are similar to those observed in age-matched healthy control subjects [10], [13]. Rodent studies indicate that muscle IR is primarily due to fat overconsumption and lipid accumulation, rather than mitochondrial dysfunction [14]. These findings complement studies in which skeletal muscle from C57Bl/6J mice fed a HFD for 5- or 20-weeks had increased mitochondrial fatty acid oxidative capacity, higher activity of oxidative phosphorylation enzymes, and elevated protein expression of PGC1α and mitochondrial respiratory chain subunits with concomitant impairment in glucose clearance and insulin action [15]. In contrast, it has been reported that mRNA levels of oxidative phosphorylation genes in mice decreased due to HFD [16]. Whether mitochondrial function is elevated, reduced, or maintained due to HFD, and whether mitochondrial biogenesis or impairment occurs is not clear.

Skeletal muscle mitochondrial function can be measured *in vitro* employing several methods including oxidative enzyme activity assays [17], [18], [19], [20], quantifying mRNA and oxidative phosphorylation associated proteins [7], [8], [18], [21], [22], and evaluating mitochondrial content, morphology, and respiration [19], [22], [23]. Here we describe the application of a chemical probe-based approach capable of broadly profiling the functional content of nucleotide binding proteins in skeletal muscle mitochondria. We used this method to identify and quantify ATP-binding proteins with altered abundance or activity in a mouse model of diet-induced obesity, IR, and T2DM over 2-, 8-, and 16-weeks of HFD feeding. Our findings argue against the concept that IR is mediated by a functional deficiency of oxidative enzymes in skeletal muscle mitochondria; but rather that mitochondrial oxidative and lipid metabolism functions for HFD fed mice are similar or elevated relative to SD fed mice.

## Materials and Methods

### Animals

Adult male C57Bl/6J mice were purchased from Jackson Laboratory (Sacramento, CA). Mice were purchased at 6-, 12-, and 20-weeks of age; having been on the standard and high-fat diets for 0-, 6-, and 14-weeks, respectively. Six mice per condition, per time-point, for a total of 36 mice were used for the study. The animals were housed individually in a temperature- and humidity-controlled room (23±1°C and 55±10%, respectively) on a 12-hour light/dark cycle with free access to food and water. Mice were fed *ad libitum* for a period of two weeks with a standard lab diet (SD) (10% calories from fat, 20% calories from protein, 70% calories from carbohydrates, 3.85 kcal/g; Research Diets D12450Bi, New Brunswick, NJ) or with a high-fat diet (HFD) (60% calories from fat, 20% calories from protein, 20% calories from carbohydrates, 5.24 kcal/g; Research Diets D12492i). All animal experiments were conducted in strict accord with institutional guidelines for the care and use of laboratory animals. The Battelle Richland Institutional Animal Care and Use Committee approved protocol #2010-45.

### Isolation of skeletal muscle mitochondria

Mitochondria were isolated from skeletal muscle tissue employing a method adapted from Frezza et al [24]. Mice were euthanized by CO_2_ asphyxiation and the hind leg skeletal muscle was rapidly harvested, suspended in ice cold PBS (1x, pH 7.4∶11.9 mM phosphates, 137 mM NaCl, 2.7 mM KCl) (5 mL) supplemented with EDTA (10 mM), and minced with a razor blade. The minced tissue was rinsed twice with cold PBS (5 mL) supplemented with EDTA (10 mM), and then suspended in a freshly prepared trypsin solution (10 mL; 0.25% trypsin EDTA solution (10 mL) into PBS (90 mL)). The skeletal muscle-trypsin EDTA solution was incubated on ice 30 minutes, centrifuged at 200× *g* for 5 minutes, and the supernatant discarded. Mitochondrial isolation buffer 1 (10 mL; 67 mM sucrose, 3 mM EGTA, 50 mM KCl, 10 mM EDTA, and 1% bovine serum albumin, pH 7.4) was added to the pellet. The pellet was homogenized using a mechanical Teflon pestle (10 passes, 1,600 rpm). The homogenized solution was dounce homogenized with pestle A (10 passes) and pestle B (10 passes). Skeletal homogenate was kept cold between procedures to minimize proteolytic degradation. The homogenate was centrifuged at 700× *g* for 10 minutes at 4°C. The supernatant was transferred to glass centrifuge tubes and centrifuged at 8,000× *g* for 10 min at 4°C. The supernatant was decanted, and the mitochondrial pellet was resuspended in ice-cold mitochondrial isolation buffer 2 (5 mL; 250 mM sucrose, 3 mM EGTA, and 10 mM Tris-HCl, pH 7.4). Mitochondria were lysed by probe sonication, and Bradford protein concentration assays were obtained.

### Citrate synthase enzyme activity assay

We used a Citrate Synthase Assay Kit (Sigma-Aldrich) to measure the citrate synthase catalyzed reaction of acetyl-CoA and oxaloacetate to form citric acid. We followed the procedure and used 8 uL of 1 mg/mL protein in PBS. A SpectraMax spectrophotometer set at 412 nm made measurements at 10 second intervals for a 90 second duration.

### Cytochrome c oxidase enzyme activity assay

We used a Cytochrome c Oxidase Assay Kit (Sigma-Aldrich) to measure the decrease in absorbance at 550 nm of ferrocytochrome c caused by its oxidation to ferricytochrome c by cytochrome c oxidase. We followed the procedure and used 8 uL of 1 mg/mL protein in PBS. A SpectraMax spectrophotometer set at 550 nm made measurements at 10 second intervals for a 90 second duration.

### Synthesis of ATP-Activity-Based Probe (ATP-ABP) (Figure****1)

#### Hex-5-ynoic (isobutyl carbonic) anhydride (3)

To a 0°C solution of 5-hexynoic acid (**1**) (500 µL, 4.4 mmol) in tetrahydrofuran (THF) (4.4 mL) was added N-methylmorpholine (NMM) (600 µL, 5.5 mmol) and isobutyl chloroformate (**2**) (660 µL, 5.1 mmol). The reaction was maintained at 0°C for 1 hour. A white precipitate was filtered, and the filtrate concentrated by rotary evaporation. The residue was dissolved in diethyl ether (3 mL), and washed with H_2_O (3 mL). The organic layer was dried over Na_2_SO_4_, filtered, and concentrated to give **3** (903 mg, 97%) as a clear oil. ^1^H-NMR (500 MHz, CDCl_3_) δ: 4.08 (d, J = 1.5 Hz, 2H), 3.75 (s, 1H), 2.65 (t, J = 7 Hz, 2H), 2.32 (td, J = 2.5 Hz, 4.5 Hz, 2H), 2.05−1.86 (m, 4H), 0.97 (d, J = 6.5 Hz, 6H).

**Figure 1:**
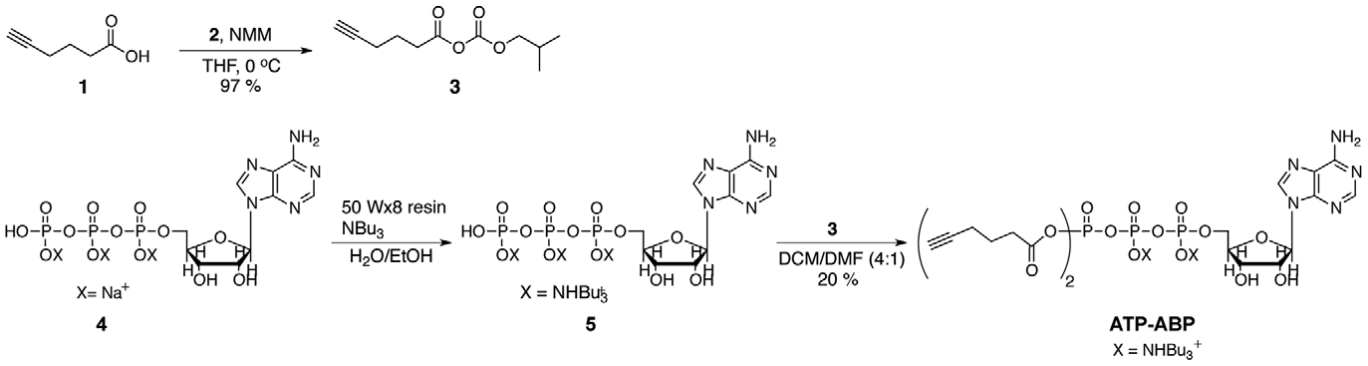
Synthesis of ATP-ABP.

#### Adenosine-5′ Triphosphate (ATP) di-N-tertbutylammonium salt hydrate (5)

ATP disodium salt hydrate (**4**) (2.23 g, 5 mmol) was dissolved in deionized H_2_O (50 mL). To a column was added strongly acidic Spectra Analytical 50 WX-8 resin (8 cm depth). The resin was washed with deionized H_2_O until the pH of the effluent was neutral, then **4** was added to the column. The effluent from the column was dripped into a 0°C stirred solution of tri-n-butylamine (2.38 mL, 10 mmol) in ethanol (EtOH) (20 mL). Deionized H_2_O was added to the column and collected in the solution of tri-n-butylamine. This continued until the effluent was neutral. EtOH (3×10 mL) was added, and the volatiles removed by rotary evaporation. Dry N,N-dimethylformamide (DMF) (10 mL) was added with molecular sieves (4 Å activated) to yield a 0.45 M solution of **5**. P^31^-NMR (236 MHz, D_2_O) δ: −7.77 (d), −8.30 (d), −20.13 (t).

**Figure 2:**
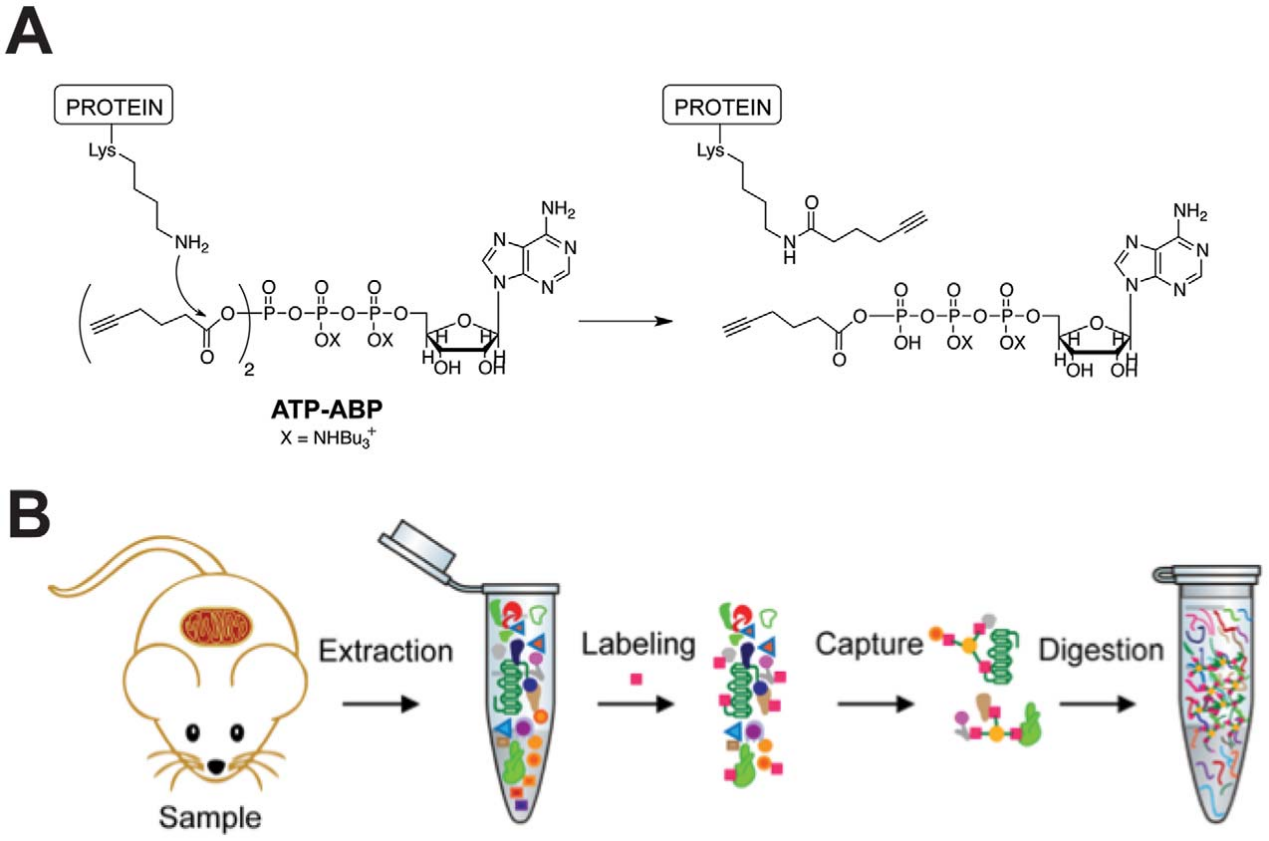
Activity-based protein profiling of skeletal muscle mitochondria with ATP-ABP. (A) Probe structure of **ATP-ABP**, and reactivity with ATPases and ATP-binding proteins. The ε-amino group of lysine reacts with the mixed anhydride of **ATP-ABP** to form a stable amide bond on the protein to a six-carbon moiety containing the click chemistry (CC) compatible alkyne. Subsequent CC reaction to biotin-azide permits enrichment of **ATP-ABP**-labeled proteins. (B) Sample preparation of **ATP-ABP** labeled skeletal muscle mitochondrial proteins. Mitochondria were extracted from skeletal muscle tissue from individual mice, labeled with the probe (pink square), captured on streptavidin agarose resin (yellow circle), digested on-resin with trypsin, and the subsequent peptides were measured by LC-MS/MS.

#### Adenosine-5′ di-hexyn-acyl-triphosphate di-N-tertbutylammonium salt hydrate (ATP-ABP)

To a 0.1 M solution of **5** (0.5 mmol) in anhydrous dichloromethane (DCM)∶DMF (4∶1, 5 mL) was added a solution of anhydride **3** (319 mg, 1.5 mmol) in anhydrous DCM/DMF (4∶1, 5 mL) at room temperature. The resulting solution stirred overnight, and then was diluted with DCM and extracted with H_2_O. The aqueous phase was removed by lyophilization yielding a crude product that was purified on a Reveleris medium-pressure liquid chromatography system (Grace Davison Discovery Sciences, Deerfield, IL) using a C18 reverse phase 12 g cartridge (100% H_2_O to 100% MeOH) to yield **ATP-ABP** (18.8 mg, 4%). P^31^-NMR (236 MHz, D_2_O) δ:−8.59, −16.87, −20.72.

### *In vitro* probe labeling for mass spectrometry measurement

Skeletal muscle mitochondrial lysate proteomes (1 mL, 1 mg/mL protein in PBS) were treated with **ATP-ABP** (20 µM) and incubated for 1 hour at 37°C on a thermal mixer with mild agitation. Following probe incubation, proteomes were treated with biotin-azide [25] (36 μM), tris(2-carboxyethyl) phosphine (TCEP) (250 µM, freshly prepared in water), tris[(1-benzyl-1*H*-1,2,3-triazol-4-yl)methyl]amine (TBTA) (50 µM, prepared in 4∶1 tert-butanol∶DMSO), and CuSO_4_ (0.50 mM, prepared in water). The samples were vortexed and incubated at room temperature in the dark for 1.5 hours. At this point, proteins precipitated from solution.

The samples were centrifuged at 11,600× *g* for 4 minutes at 4°C. The supernatant was discarded, and 400 μL of ice-cold methanol was added to the pellet. The samples were probe sonicated while on ice. The samples were vortexed and rotated for 10 minutes at 4°C. The samples were centrifuged again at 11,600× *g* for 4 minutes at 4°C, and the supernatant discarded. Ice-cold methanol was added again, and the sonication, rotation, and centrifugation repeated. The supernatant was discarded, taking care to remove as much residual methanol as possible. The samples were removed from the ice and left to air-dry for 5 minutes at room temperature (∼24°C). The samples were then dissolved in 1.2% SDS in PBS (520 μL) and probe sonicated. The samples were heated at 95°C on a thermal mixer with moderate agitation. The samples were then centrifuged at room temperature at 6,000× *g* for 4 minutes. A BCA assay was performed to determine sample concentration.

For enrichment of probe-labeled proteins, a 100 μL aliquot of streptavidin agarose resin (Thermo Fisher Scientific, Rockford, IL; 1–3 mg biotinylated BSA protein per mL resin) per prepared sample was placed in a Bio-Spin Chromatography Column (Bio-Rad, Hercules, CA) on a vacuum manifold. The resin was washed 3×1 mL with PBS, and transferred to a 15 mL vial using two 0.5 mL aliquots of PBS. An additional 1.0 mL of PBS was added to each tube followed by 300 μg of protein (in 1.2% SDS in PBS). The normalized protein loading onto streptavidin results in an analysis that evaluates protein function on a per unit mass basis. The total volume of each tube was set to 3.0 mL, giving a final SDS concentration of 0.2%. The tubes were rotated end-over-end for 4 h at room temperature.

Following streptavidin capture of **ATP-ABP**-labeled proteins, the solution in the falcon tubes was transferred into the Bio-Spin columns, and the solution removed. The resin was then washed with 0.5% SDS in PBS (1 mL, repeat 3×), 6 M urea in PBS (1 mL, repeat 3×), MilliQ water (1 mL, repeat 3×), and PBS (1 mL, repeat 5×). The resin was transferred to sealed 1.5 mL tubes using two 0.5 mL aliquots of PBS. The samples were centrifuged at 6,000× *g*, and the supernatant discarded.

To reduce the disulfide bonds of the enriched proteins, 6 M urea in PBS (400 μL) was added to the resin in the tubes, followed by 20 μL of 100 mM TCEP. This mixture was placed on the thermal mixer at 37°C with strong agitation for 30 minutes. To alkylate the reduced sulfhydryl groups, 20 μL of 200 mM iodoacetamide was added directly to the mixture, and the solution was placed on the thermal mixer at 50°C with strong agitation for 45 minutes. Following alkylation, the resin was transferred back into the Bio-Spin column and washed with PBS (1 mL, repeat 9×), followed by NH_4_HCO_3_ (25 mM, pH 8; 1 mL, repeat 5×). The resin was then transferred to a low-binding 1.5 mL vial using two 500 μL aliquots of NH_4_HCO_3_. The tubes were then centrifuged at 6,000× *g* for 4 minutes, and the supernatant discarded.

To obtain peptides for MS analysis, NH_4_HCO_3_ (200 μL) was added to the resin for each sample, along with trypsin solution (2 μL; trypsin was reconstituted in 40 μL of NH_4_HCO_3_). The resin solutions were placed on the thermal mixer at 37°C with strong agitation for 15 hours. Following trypsin digestion, the tubes were centrifuged at 6,000× *g* for 4 minutes, and the supernatant was carefully collected. To the resin was added NH_4_HCO_3_ (150 μL), and the tubes were placed on a thermal mixer at 37°C with strong agitation for 10 minutes. Again, the tubes were centrifuged at 6,000× *g* for 4 minutes, and the supernatant was carefully collected and added to the prior collection. The volatiles were then removed from the combined tryptic peptide supernatant using a speed vacuum. The dried peptides were reconstituted in NH_4_HCO_3_ (40 μL), and heated for 10 minutes at 37°C with mild agitation. To remove any solid particulates, the samples were centrifuged at 100,000× *g* for 20 minutes at 4°C. From each ultracentrifuge vial was removed 25 μL for subsequent MS analysis. Samples were stored at −20°C until analysis.

### LC-MS analysis of enriched mitochondrial proteins

Peptides were separated by high resolution, reversed phase capillary liquid chromatography as previously described [26]. Briefly, MS analysis was performed using a ThermoFinnigan LTQ-Orbitrap MS (Thermo Scientific, San Jose, CA) outfitted with a custom ion funnel and electrospray ionization (ESI) interface. Data was acquired for 100 min, beginning 65 min after sample injection (15 minutes into gradient). Orbitrap spectra were collected from 400–2000 m/z at a resolution of 100 k, followed by data-dependent ion trap MS/MS spectra of the six most abundant ions using a 35% collision energy. A dynamic exclusion time of 30 seconds was used to discriminate against previously analyzed ions.

### Data analysis

LTQ-MS raw data was extracted using Extract_MSn (version 3.0), and analyzed with the SEQUEST algorithm (V27, revision 12) searching the MS/MS data against the NCBI mouse protein database. Data filtering criteria based on the MS-GF score [27] and precursor ion mass accuracy (+/−10 ppm) and were used to limit false positive identifications to <1% at the peptide level estimated employing a reverse database search [28]. Relative protein abundance was estimated employing the accurate mass and time tag strategy [29]. Analysis of quantification of the changes in protein abundance between SD and HFD mice was performed and visualized using DAnTE [30]. Briefly, peptide intensities from the LC-MS analyses were log_2_ transformed, and peptides exhibiting significant differences (p<0.05) in abundance were identified applying an analysis of variance (ANOVA) test. Peptide abundances were then “rolled up” to the unique protein level employing the R-rollup method implemented in DAnTE. Functional enrichment and pathway analysis was performed with Ingenuity Pathways Analysis (Ingenuity® Systems).

## Results

We designed **ATP-ABP** to target kinases, ATPases, and nucleotide-binding proteins that use ATP as a binding group scaffold. The **ATP-ABP** has two reactive acyl phosphate groups for capturing probe-protein interactions. In an effort to reduce the probe size to minimize steric interference and increase protein active and binding site inclusion, we incorporated reactive acyl phosphate moieties, which directly acylate lysine ε-amino groups of ATPases and ATP- and nucleotide-binding proteins. The incorporation of alkyne units in the probe, and their subsequent transfer to the protein upon probe-protein reaction, allows post-labeling conjugation of reporter groups such as fluorophores or biotin for affinity enrichment via click chemistry (CC). The bio-compatible CC reaction [25] is a copper-catalyzed cycloaddition between an azide-bearing molecule and an alkyne-bearing ABP.

### Skeletal muscle mitochondria activity-based protein profiling – proteome coverage

**Figure 3:**
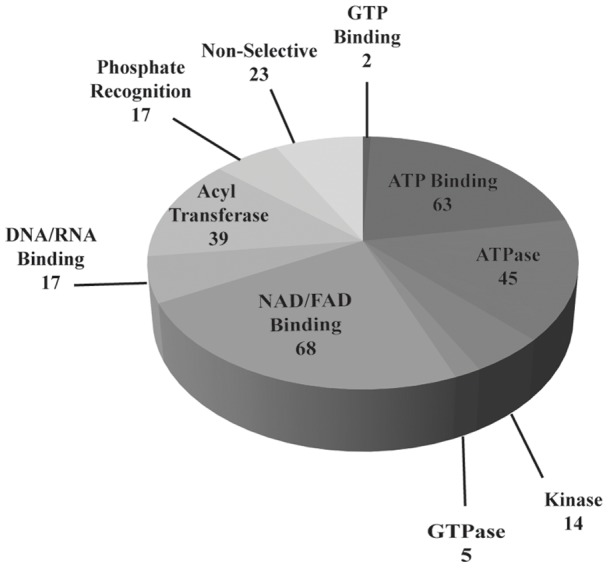
ATP-ABP labeling profile of skeletal muscle mitochondrial lysates from HFD and SD fed mice. 293 total high confidence unique proteins were identified in total.

Isolated skeletal muscle mitochondria from high-fat (HFD) and standard diet (SD) fed mice were lysed and labeled with **ATP-ABP** allowing us to identify and quantify ATP binding mitochondrial proteins. To directly assess the effect of the HFD on skeletal muscle mitochondrial enzyme function, we labeled skeletal muscle mitochondrial lysates from mice fed HFD and SD for 2-, 8-, and 16-weeks. Following protein probe labeling, biotin was covalently appended to probe-labeled proteins by CC (**Figure** **2A**), allowing streptavidin agarose based enrichment of probe-labeled proteins. Proteins were identified by LC-MS/MS analysis of tryptic peptides derived from on bead digestion of captured biotinylated probe labeled proteins (**Figure** **2B**) and quantified employing the accurate mass and time (AMT) tag strategy [29]. Evaluating across the three time points and both diet conditions resulted in the confident identification of 293 proteins (**Table** **S1**). Of the 293 distinct probe-labeled proteins identified by LC-MS analysis, ∼92% are classifiable as having an ability to recognize a structural element of the probe (**Figure** **3**). This includes ATP phosphohydrolases, represented by 45 ATPases, 14 kinases, and 5 GTPases. The probe labeled 63 ATP- and two GTP-binding proteins, and 68 NAD- or FAD-binding proteins. Additionally, 39 proteins known to bind or react with acetyl-CoA or similar CoA molecules were labeled. This reactivity is likely governed by adenine recognition and hydrolysis of the mixed anhydride portion of **ATP-ABP**, which is similar to the hydrolysis of the common target acetyl-CoA. The 39 acetyl-CoA binding proteins include 11 acetyl-CoA dehydrogenases that require ATP or FAD-binding for function. Recognition of the adenine portion of **ATP-ABP** is likely responsible for labeling 17 DNA or RNA-binding proteins. The probe also labeled 17 proteins that recognize phosphate; several of these hydrolyze non-nucleotide phosphate, such as pyridoxal phosphate. The remaining 23 labeled proteins have no known nucleotide binding capability, but do contain surface lysines that were most likely non-selectively acylated by the probe [31].

### Canonical pathway analysis of probe-labeled proteins in skeletal muscle mitochondria

Analysis of functional enrichment was performed on the full complement of proteins identified regardless of diet or age. As shown in **Table 1**, pathway analysis (Ingenuity Pathway Analysis) revealed that many of the probe-labeled proteins are directly involved in oxidative phosphorylation (OXPHOS), mitochondrial dysfunction, and lipid metabolism (the full list of proteins and assigned annotations can be found in **Table S2**). The mitochondrial oxidative phosphorylation pathway was significantly represented in probe-labeled proteins; 59 of 146 oxidative phosphorylation proteins were identified. This includes 29 NADH dehydrogenases from the Complex I ubiquinone biosynthesis pathway of oxidative phosphorylation, three succinate dehydrogenases from Complex II, eight proteins from the cytochrome *bc_1_* complex (III) including two cytochrome *c* reductases, eight cytochrome *c* oxidases from complex IV, and 10 mitochondrial ATP synthase subunits of complex V. The majority of these proteins are also assigned to the mitochondrial dysfunction pathway; 57 of 169 proteins associated with mitochondrial dysfunction were identified with **ATP-ABP**. Additional proteins associated with this pathway include AIFM1, GPD2, HSD17B10, OGDH, PDHA1, and PRDX3.

**Table 1:** Ingenuity Pathway Analysis of 293 proteins labeled by **ATP-ABP** in mouse skeletal muscle mitochondria lysates from HFD and SD fed mice for 2-, 8-, and 16-weeks.

Canonical Pathway	p-value	Ratio
Oxidative Phosphorylation	2.63E-07	59/146
Mitochondrial Dysfunction	5.50E-07	57/169
Ubiquinone Biosynthesis	1.90E-04	33/105
Valine Leucine and Isoleucine degradation	1.62E-03	26/106
Propanoate Metabolism	3.09E-03	23/121
Citrate Cycle	6.31E-03	17/57
Butanoate Metabolism	1.38E-02	19/126
Fatty Acid Metabolism	2.40E-02	24/171
Glycolysis/Gluconeogenesis	2.63E-02	19/120
Pyruvate Metabolism	3.23E-02	17/132

The *p*-value indicates the significance of pathway enrichment. The ratio is the number of proteins identified by probe-labeling to the number of proteins assigned to that pathway by IPA.

### Mitochondrial protein functions regulated by diet over time

**Figure 4:**
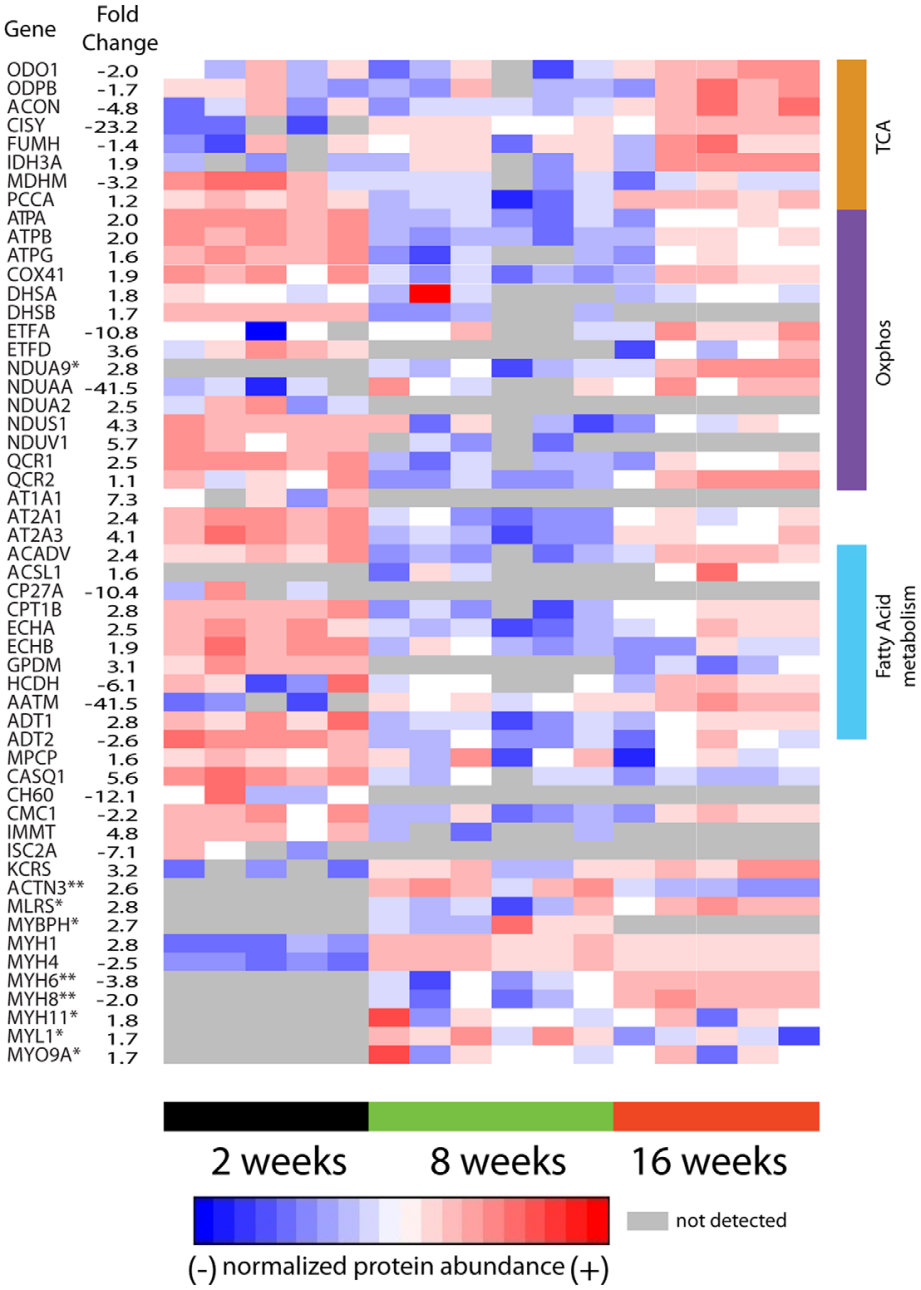
Diet induced alterations in protein abundance. Shown are proteins (rows) found to significantly differ in abundance (p<0.05) comparing mice (columns) on HFD and SD for 2-, 8-, or 16-weeks. The average fold change between mice on SD and HFD is reported for the 2 week time interval unless otherwise indicated, * indicates fold change at 8 weeks, and ** indicates fold change at 16-weeks. A single outlier at 2- and 16-week time points was removed from the HFD group.

Quantitative differences in peptide/protein abundance were determined using the accurate mass and time (AMT) tag approach [29]. Comparison of abundances of probe labeled proteins isolated from mice on HFD and SD at 2-, 8-, and 16-weeks resulted in identification of significant differences (*p*-value ≤0.05) in inferred protein abundance. There were 54 proteins identified as having significantly altered abundances due to diet (**Figure** **4**).

We evaluated mitochondrial function with **ATP-ABP** by analyzing mitochondrial skeletal muscle lysates on a per unit mass basis. We found a rapid response in which the vast majority of diet related differences in protein abundance occurred following 2-weeks on a HFD compared to SD. Following two weeks on HFD proteins associated with the TCA cycle were less functional with 7/9 proteins associated with TCA cycle reduced 1.7-fold or greater, with citrate synthase being reduced ∼23-fold for HFD compared to SD mice. However, 11/15 proteins involved in oxidative phosphorylation had higher functional activity in HFD fed mice at 2-weeks. Proteins associated with mitochondrial dysfunction are largely increased (11/16) in abundance following 2-weeks on a HFD relative to SD. Proteins from all five respiratory chain subunits had increased **ATP-ABP** labeling, and several proteins associated with fatty acid metabolism were up-regulated in the HFD fed mice.

Increased duration (>8 weeks) on the HFD results in differences in mitochondrial function becoming negligible. The rapid initial response to diet was followed by adaptation and return to near normal function. At 16-weeks on the HFD proteins with annotated functions associated with OXPHOS, mitochondrial dysfunction, and fatty acid metabolism were elevated compared to SD fed mice. Proteins associated with OXPHOS, the mitochondrial respiratory chain, and fatty acid metabolism had the highest function at 2-weeks, the lowest function at 8-weeks, and moderate function at 16-weeks. In contrast, TCA cycle proteins tended to have lowest function at 2-weeks, elevated function at 8-weeks, and highest function at 16-weeks.

Overall the data show an adaptive response by HFD fed mice at 2-weeks, followed by mitochondrial function not widely different from SD fed mice at 8- and 16-week time intervals.

### Oxidative capacity measured by citrate synthase activity

**Figure 5:**
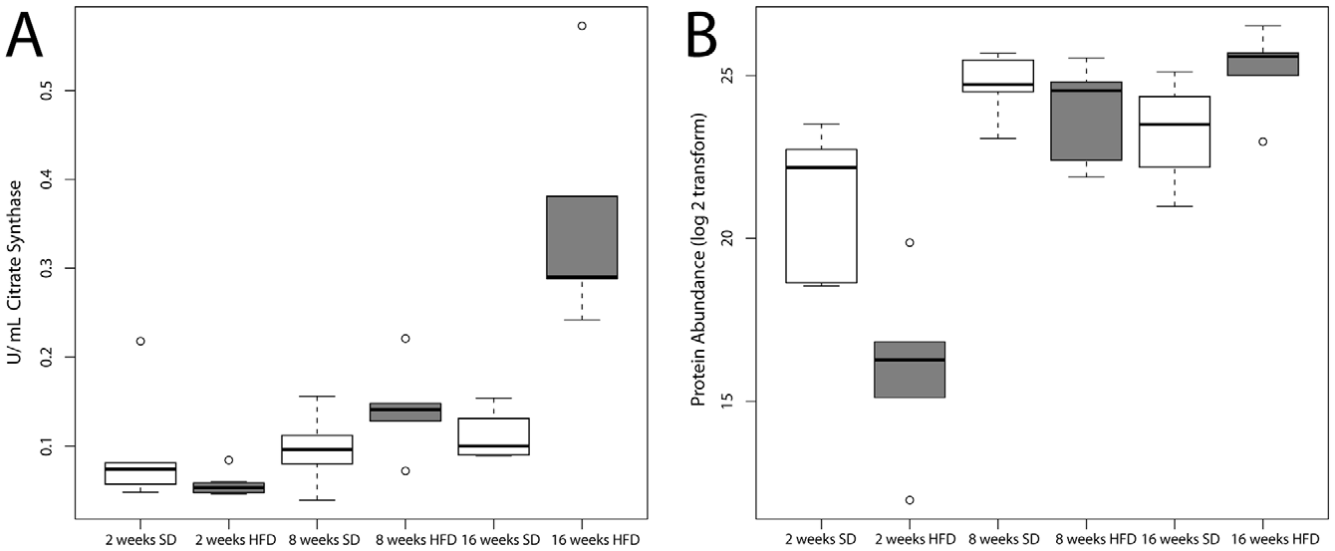
Comparison of citrate synthase activity measured by an activity assay and by LC-MS based ABPP using ATP-ABP . (A) The citrate synthase activity was measured for each skeletal muscle mitochondrial lysates from individual mice. Results show the mean +/− standard error. (B) Protein abundance identified by **ATP-ABP** labeling of citrate synthase. Results show the mean of the abundance measured by MS +/− standard error. A single outlier at 2- and 16-week time points was removed from the HFD group. Each box in (A) and (B) contains all measured data for each point. The line within the box indicates the mean, and capped dashed lines represent the standard error from the mean.

We measured citrate synthase (CS) activity in the lysates of skeletal muscle mitochondria from all individual SD and HFD fed mice to validate our ABPP results via an alternative oxidative capacity assay. Measurement of CS activity is an established method for quantifying mitochondrial oxidative enzyme activity [6]. Consistent with the ABPP mass spectrometry-based proteomic results of skeletal muscle mitochondria, CS activity is reduced in HFD compared to SD mice at week 2, elevated to the same extent in both HFD and SD at week 8, and increased in HFD versus SD mice at week 16 (**Figure** **5**). The CS activity assay shows a larger change for mice on a HFD at 16-weeks than the proteomics data, but consistent trends. This trend is also true to the proteomic patterns showing that nearly all of the probe-labeled proteins associated with the TCA cycle increase in abundance with increased duration of both the HFD and SD. Importantly, the probe-labeling experiment involves direct irreversible binding of the probe to CS, whereas the CS assay is a measurement of substrate turnover.

### Measurement of cytochrome C oxidase activity

**Figure 6:**
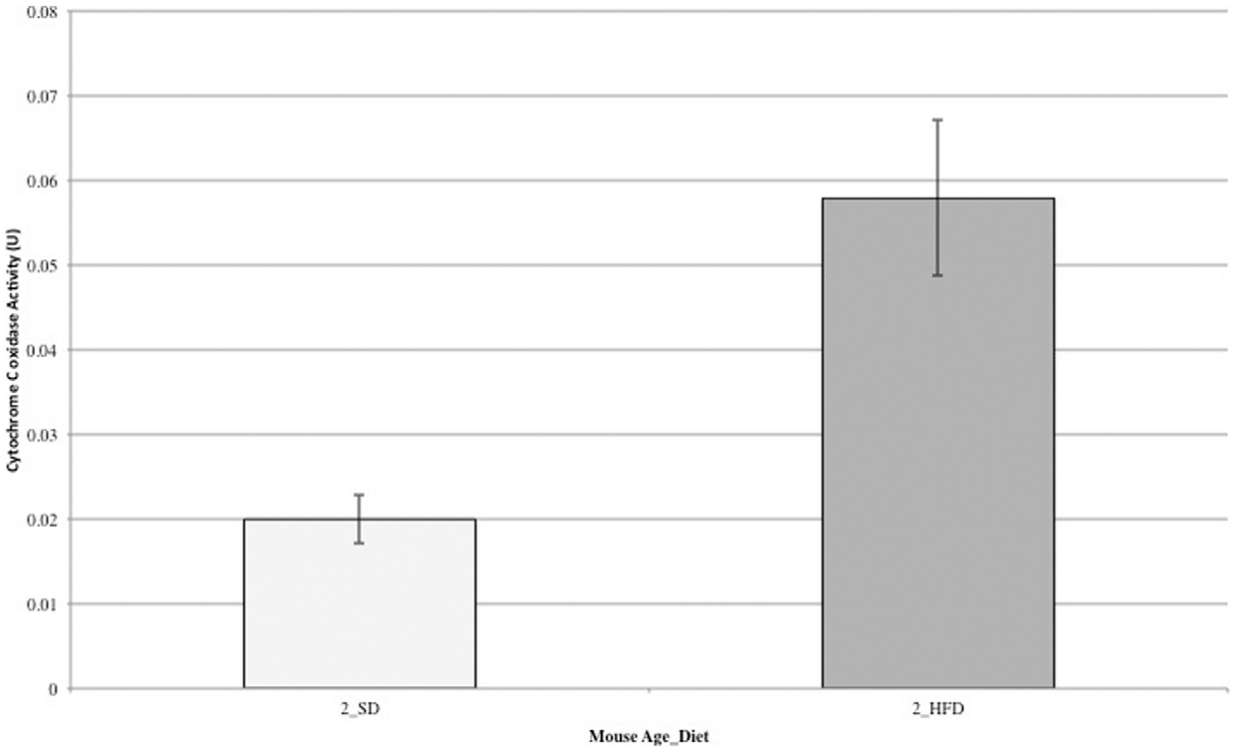
Comparison of cytochrome C oxidase activity between HFD- and SD-fed mice at two weeks, measured by an absorbance assay. The cytochrome C oxidase activity was measured for each skeletal muscle mitochondrial lysate from individual mice. Results show the mean +/− standard error. A single outlier for both HFD- and SD-fed mouse groups was removed. The fold change closely correlates with the fold change for COX41 measured by **ATP-ABP** (**Figure 3**).

In a second validation experiment of our ABPP results, we measured cytochrome C oxidase activity in week two HFD – and SD-fed mice by an absorbance based assay (Sigma Aldrich) (**Figure** **6**), and compared to the fold change reported in **Figure** **4**. Using our ABPP approach, the enzyme, COX41, was found to be 1.9-fold upregulated in function when comparing HFD- to SD-fed mice at week two of the diets. The absorbance-based assay reveals an approximate 3-fold increase. The ABPP approach is in close agreement with the absorbance-based assay, and accounting for the standard error of the absorbance assay measurements, the ABPP approach is nearly identical.

## Discussion

We employed an activity-based protein profiling (ABPP) approach, which is a young but established technology for functionally profiling classes of proteins within complex biological systems [32], [33]. ABPP uses activity-based probes (ABPs), which are small-molecule active site and binding site-directed synthetic probes, to specifically enrich probe targets for downstream LC-MS analysis. This is a significant advantage over traditional non-targeted proteomic measurements of skeletal muscle tissue that suffer from undersampling due to high abundance of cytoskeletal and contractile proteins.

We employed **ATP-ABP,** a new CC-compatible ATP-derived probe, to label mitochondrial lysates of skeletal muscle mitochondria from high fat and standard diet fed C57Bl/6J mice. The probe targeted kinases and ATPases likely coupling through binding site lysine residues. The probe labeling mechanism involves amino acid acylation or transfer of the pendant alkyne from the probe to the protein upon nucleophilic attack of the mixed carboxylic phosphoric anhydride by a protein amino acid. Copper-catalyzed CC attachment of chromogenic or fluorogenic tags, or enrichment moieties such as biotin enable downstream analysis. Additionally, the small alkyne group reduces the overall size of the probe thereby increasing the inclusion of the probe into ATP-binding sites within proteins.

Our ABPP approach employing the **ATP-ABP** broadly covered ATP-dependent proteins and simultaneously provided profiles for numerous proteins from pathways critical to the study of diabetes, such as mitochondrial dysfunction, oxidative phosphorylation, and lipid metabolism. We chose to label lysates at 2-, 8-, and 16-weeks given that following 5 weeks on a HFD a significant impairment in glucose clearance, and impaired insulin action in rodent skeletal muscle was previously reported [15].

Feeding mice high-fat diets results in an early increase in mitochondrial oxidative phosphorylation enzyme activity and lipid metabolism, and a decrease in abundance of TCA cycle associated enzymes. However, over time mitochondrial probe-labeling activity in HFD fed mice was similar to, or slightly higher than, activity observed in SD fed mice. This observed “normal” mitochondrial ATP-dependent function is likely concomitant with development of insulin resistance and increased intramuscular lipids, as previously reported [15], [34], [35]. Of critical note, these measurements represent activity measured per unit mass mitochondria, i.e. protein reactivity with **ATP-ABP** per unit mass, and not mitochondrial abundance within the skeletal muscle. Our ABPP derived findings contradict earlier studies that implicate high-fat diets in leading to altered oxidative phosphorylation transcript abundance in the prediabetic/insulin-resistant and diabetic states [7], [16], [18], [19], [23]. Our study is consistent with the concept that IR is not a direct result of mitochondrial deficiency, given that the mitochondrial proteins recover and show little difference comparing mice on HFD and SD following 16-weeks.

A number of prior studies have shown that high-fat diets leading to intramuscular lipid accumulation result in impaired glucose clearance and insulin resistance, but do not negatively impact mitochondrial function [10], [11], [12], [13], [14], [36], [37], [38], [39]. Recently, investigators found that muscle insulin resistance is a case of fat overconsumption rather than mitochondrial dysfunction [14]. Complementary to this, Turner et. al., reported that rodent skeletal muscle mitochondria at either 5- or 20-weeks on a HFD increased mitochondrial enzyme activity, fatty acid oxidative capacity, and elevated protein expression of mitochondrial respiratory chain units [15]. Studies in human muscle from patients with T2DM and muscle from obese women and normal control individuals found mitochondrial content in both T2DM and obese patients was reduced, but the remaining mitochondria maintain normal function [10], [14]. Our ABPP findings clearly demonstrate the mitochondrial oxidative phosphorylation and lipid metabolism pathways are maintained at similar functional levels between SD and HFD fed mice following 16-weeks on a HFD. We provide evidence that mammals can adapt to dietary nutrient variations and can compensate metabolically [5], [40].

Determining whether mitochondrial function is reduced or elevated, without regard for increased or decreased mitochondrial abundance, warranted further investigation. It is evident that most studies identify mitochondrial dysfunction as primarily the result of a decrease in mitochondrial abundance or altered morphology, or that increased mitochondrial activity of individuals on a HFD is due primarily to mitochondrial biogenesis. It has been reported that there is an approximate 30% reduction in mitochondrial content in the muscles of insulin-resistant individuals when compared to control subjects, and this too has been described as mitochondrial dysfunction [4], [7], [8], [19], [41]. However, the remaining mitochondria function at the same levels as normal control individuals [10]. Conversely, other studies have reported mitochondrial biogenesis as a rescue response to high-fat feeding, and the increased number of mitochondria result in elevated oxidative phosphorylation and lipid metabolism [12], [15]. Our experimental findings provide evidence that ATP-dependent mitochondrial function in mice fed HFD function at levels akin to control mice fed the SD, and short-term 2-week HFD feeding increases several proteins aligned with oxidative phosphorylation and lipid metabolism. Since mitochondrial activity was measured per unit mass of mitochondria instead of on total mitochondrial content, activity was found to be approximately equivalent in HFD and SD fed, regardless of mitochondria abundance.

Mitochondrial oxidative metabolism of glucose and lipids creates NADH or FADH_2_ generated from glycolysis, the TCA cycle, and β-oxidation. Oxidation of NADH and FADH_2_ subsequently generates an electrochemical gradient across the inner mitochondrial membrane (complexes I, II, and IV) that drives ATP synthase (complex V) to produce ATP from ADP [3]. The mitochondria elegantly orchestrates fatty acid oxidation, the TCA cycle, and the electron transport chain to optimize ATP production to meet cellular energy needs [5], [42], [43]. It has previously been shown that the metabolic efficiency of skeletal muscle mitochondria isolated from different skeletal muscle fiber types in response to a high-fat diet varies with regard to fatty acid oxidation [44]. A targeted metabolomic screening showed that a HFD increased the rate of fatty acid oxidation but depleted TCA organic acid intermediates, thereby rendering the TCA cycle limited in the completion of oxidative phosphorylation [45]. This in turn leads to reduced glucose oxidation and insulin resistance. Interestingly, our data indicates that mice fed high-fat diets for 2-weeks have reduced TCA cycle function but increased β-oxidation (e.g., trifunctional mitochondrial protein) and mitochondrial respiratory chain activity (all complexes) when compared to mice fed standard diets for 2-weeks (**Figure** **4**). This likely represents initial adaptation to the increased lipid availability. Our findings corroborate previous studies that show elements of mitochondrial dysfunction and insulin resistance at early time points in mice fed HFDs [16]. Importantly, at the 8- and 16-week time points this discrepancy in pathways associated with mitochondrial energy metabolism is no longer evident.

In summary we have utilized ABPP to evaluate proteins in pathways associated with mitochondrial oxidative phosphorylation, lipid metabolism, and mitochondrial dysfunction in mice fed high-fat or standard control diets. We found that per unit mass of mitochondria proteins associated with oxidative phosphorylation and lipid metabolism had very little variation between HFD and SD fed mice, except at the earliest time point, which likely represents acclimatization to the high-lipid content nutrient supply. These findings support prior evidence showing that mitochondrial dysfunction is not directly caused by high-fat diets. Prior mouse model studies showed that mice on long-term high-fat diets developed insulin resistance as early as 5-weeks. Though not measured directly in our study, the high-fat fed mice at 8- and 16-weeks likely developed IR, thus our findings indicate no evidence of per unit mitochondrial dysfunction. We conclude that IR is neither cause nor consequence of mitochondrial dysfunction. Finally by the end-point in our study, HFD mice had TCA cycle, β-oxidation, and respiratory chain proteins at levels similar to or higher than standard diet fed mice, further supporting evidence that per unit mass mitochondria in mice on high-fat diets increase mitochondrial oxidative metabolism.

## Supporting Information

Table S1 A complete list of the 293 distinct **ATP-ABP**-labeled proteins identified by LC-MS analysis.
(XLSX)

Table S2 A complete list of proteins and assigned annotations by Ingenuity Pathway Analysis.
(DOC)
