# Supplementary material for: Activity-Based Protein Profiling Reveals Mitochondrial Oxidative Enzyme Impairment and Restoration in Diet-Induced Obese Mice
Source: PLoS One. 2012 Oct 24;7(10):e47996. doi: 10.1371/journal.pone.0047996 (PMC3480472; doi:10.1371/journal.pone.0047996)
Supplement: Table S2 — A complete list of proteins and assigned annotations by Ingenuity Pathway Analysis. (DOC) [file pone.0047996.s002.doc]

|  | ATP5B | ATP5A1 | AIFM1 | ALDH6A1 | ACAA2 | ACAD10 | ACO2 (includes EG:11429) | ACADS | ACAA2 | ACSL1 |
| --- | --- | --- | --- | --- | --- | --- | --- | --- | --- | --- |
|  | ATP5C1 | ATP5A1 | COQ3 | ACAD10 | ACADL | CLYBL | ACAT1 | ACAD10 | ACSS1 | ACAT1 |
|  | ATP5F1 | ATP5B | ND1 (includes EG:140531) | ACADL | ACADM | CS | ALDH2 | ACADL | ALDH2 | ACOT9 |
|  | ATP5G1 | ATP5C1 | NDUFA10 | ACADM | ACADSB | DLD | ALDH4A1 | ACADM | ALDH4A1 | ACSL1 |
|  | Atp5h (includes EG:306478) | ATP5J | NDUFA12 | ACADS | ACADVL | DLST | AUH | ACADS | ALDOA | ACSS1 |
|  | ATP5J | COX2 (includes EG:140540) | NDUFA13 | ACADSB | ACAT1 | FH | ECH1 | ACADSB | DLAT | ALDH2 |
|  | ATP5J2 | COX4I1 | NDUFA2 | ACADVL | ACSF3 | IDH1 | ECHS1 | ACADVL | DLD | ALDH4A1 |
|  | ATP5L | COX5A (includes EG:12858) | NDUFA3 | ACAT1 | ACSL1 | IDH2 | HADH | ACAT1 | ENO1 | DLAT |
|  | ATP5O | COX6B1 | NDUFA4 | ALDH2 | ACSS1 | IDH3A | HADHA | ACSL1 | ENO3 | DLD |
|  | ATP8 (includes EG:17706) | COX6C | NDUFA5 | ALDH4A1 | ALDH2 | MDH2 (includes EG:17448) | HADHB | ACSL6 | Gapdh (includes others) | HADHA |
|  | COX2 (includes EG:140540) | COX7A1 | NDUFA6 | ALDH6A1 | ALDH4A1 | OGDH | HMGCL | ALDH2 | HSD17B10 | LDHA |
|  | COX4I1 | COX7A2 | NDUFA7 | AUH | ALDH6A1 | PC | HSD17B10 | ALDH4A1 | LDHA | MDH2 (includes EG:17448) |
|  | COX5A (includes EG:12858) | Cox8b | NDUFA8 | ECH1 | AUH | SDHA (includes EG:157074) | L2HGDH | AUH | PDHA1 | ME3 |
|  | COX6B1 | CPT1A | NDUFA9 | ECHS1 | ECH1 | SDHB | OXCT1 | CPT1A | PDHB | PC |
|  | COX6C | CPT1B | NDUFAB1 | HADH | ECHS1 | SDHC | PDHA1 | CPT1B | PDHX | PDHA1 |
|  | COX7A1 | CYC1 | NDUFB10 | HADHA | HADHA | SUCLA2 | PDHB | CPT2 | PFKM | PDHB |
|  | COX7A2 | GPD2 | NDUFB11 | HADHB | HADHB | SUCLG1 | SDHA (includes EG:157074) | GPDM | PGAM2 | PDHX |
|  | Cox8b | HSD17B10 | NDUFB4 | HIBADH | LDHA |  | SDHB | ECH1 | PGK1 | PKM2 |
|  | CYC1 | NDUFA10 | NDUFB5 | HMGCL | MUT |  | SDHC | ECHS1 | PKM2 |  |
|  | ND1 (includes EG:140531) | NDUFA12 | NDUFB7 | HSD17B10 | PCCA |  |  | HADH |  |  |
|  | NDUFA10 | NDUFA13 | NDUFB8 | IVD | PCCB |  |  | HADHA |  |  |
|  | NDUFA12 | NDUFA2 | NDUFB9 | MCCC1 | SUCLA2 |  |  | HADHB |  |  |
|  | NDUFA13 | NDUFA3 | NDUFC2 | MUT | SUCLG1 |  |  | HSD17B10 |  |  |
|  | NDUFA2 | NDUFA4 | NDUFS1 | OXCT1 |  |  |  | CP27A |  |  |
|  | NDUFA3 | NDUFA5 | NDUFS2 | PCCA |  |  |  |  |  |  |
|  | NDUFA4 | NDUFA6 | NDUFS3 | PCCB |  |  |  |  |  |  |
|  | NDUFA5 | NDUFA7 | NDUFS6 |  |  |  |  |  |  |  |
|  | NDUFA6 | NDUFA8 | NDUFS7 |  |  |  |  |  |  |  |
|  | NDUFA7 | NDUFA9 | NDUFS8 |  |  |  |  |  |  |  |
|  | NDUFA8 | NDUFAB1 | NDUFV1 |  |  |  |  |  |  |  |
|  | NDUFA9 | NDUFB10 | NDUFV2 |  |  |  |  |  |  |  |
|  | NDUFAB1 | NDUFB11 | DHSA |  |  |  |  |  |  |  |
|  | NDUFB10 | NDUFB4 | DHSB |  |  |  |  |  |  |  |
|  | NDUFB11 | NDUFB5 |  |  |  |  |  |  |  |  |
|  | NDUFB4 | NDUFB7 |  |  |  |  |  |  |  |  |
|  | NDUFB5 | NDUFB8 |  |  |  |  |  |  |  |  |
|  | NDUFB7 | NDUFB9 |  |  |  |  |  |  |  |  |
|  | NDUFB8 | NDUFS1 |  |  |  |  |  |  |  |  |
|  | NDUFB9 | NDUFS2 |  |  |  |  |  |  |  |  |
|  | NDUFC2 | NDUFS3 |  |  |  |  |  |  |  |  |
|  | NDUFS1 | NDUFS6 |  |  |  |  |  |  |  |  |
|  | NDUFS2 | NDUFS7 |  |  |  |  |  |  |  |  |
|  | NDUFS3 | NDUFS8 |  |  |  |  |  |  |  |  |
|  | NDUFS6 | NDUFV1 |  |  |  |  |  |  |  |  |
|  | NDUFS7 | NDUFV2 |  |  |  |  |  |  |  |  |
|  | NDUFS8 | OGDH |  |  |  |  |  |  |  |  |
|  | NDUFV1 | PDHA1 |  |  |  |  |  |  |  |  |
|  | NDUFV2 | PRDX3 |  |  |  |  |  |  |  |  |
|  | SDHA (includes EG:157074) | SDHA (includes EG:157074) |  |  |  |  |  |  |  |  |
|  | SDHB | SDHB |  |  |  |  |  |  |  |  |
|  | SDHC | SDHC |  |  |  |  |  |  |  |  |
|  | UQCRB | SOD2 |  |  |  |  |  |  |  |  |
|  | UQCRC1 | UQCRB |  |  |  |  |  |  |  |  |
|  | UQCRC2 | UQCRC1 |  |  |  |  |  |  |  |  |
|  | UQCRFS1 | UQCRC2 |  |  |  |  |  |  |  |  |
|  | UQCRHL | UQCRFS1 |  |  |  |  |  |  |  |  |
|  | UQCRQ | DHSA |  |  |  |  |  |  |  |  |
|  | DHSA | DHSB |  |  |  |  |  |  |  |  |
|  | DHSB |  |  |  |  |  |  |  |  |  |
